# Iron tailings rehabilitation via organic matter supplement: multifunctionality dynamics, microbial community, and enhancement mechanism

**DOI:** 10.1038/s41598-026-55048-0

**Published:** 2026-05-25

**Authors:** Heng Liu, Xiaoshan Zhang, Mingbao Liu, Feng Li, Yuanyuan Ye, Zimeng Ma, Chengfang Qiao

**Affiliations:** 1 Key Laboratory of Comprehensive Utilization of Tailings Resources of Shaanxi Province, School of Chemical Engineering and Modern Materials, Shangluo University, Shangluo, 726000 China; 2 Engineering Research Center for Mineral Resources Clean and Efficient Conversion and New Materials of Shaanxi Province, Shangluo University, Shangluo, 726000 China; 3 Qinling Ecological Environment Research Institute, Shangluo University, Shangluo, 726000 China

**Keywords:** Iron tailings, Organic amendments, Multifunctional indices, Functional microorganisms, Carbon metabolism, Biotechnology, Ecology, Ecology, Environmental sciences, Microbiology

## Abstract

Microbial communities and tailings multifunctionality are prerequisites for iron tailings’ eco-engineering into technosols, but how organic matter supplementation improves these remains unclear. This study explored multifunctional enhancement, microbial enrichment, and driving mechanisms via microcosm experiments with fulvic acid (FL), rice husk (RC), and FL + RC (FLRC). Over 60-day incubation, tailings’ multifunctional index showed exponential growth in FL treatment and pseudo-first-order kinetics in RC/FLRC, attributed to the stronger organic matter decomposition potential in FL (3 × 10^− 3^) than RC (1.4 × 10^− 4^) and FLRC (0). Network analysis revealed rare microorganisms (relative abundance < 1%, bacterial genera of *Sericytochromatia* and *Parasegetibacter*, fungal genera of *Fusarium* and *Cystobasidium*) had the strongest correlations with physicochemical factors (*r* = 0.97), indicating the rare microorganisms instead of dominant taxa drive tailings multifunctionality. Mantel tests revealed external carbon sources regulate the tailings carbon cycle via *pgk*, *cellulase*, and *pdh* genes, while the microenvironment directly propels it through *cs*, *g6pd*, *ms* and *cat* genes. Collectively, these findings clarify the key drivers regulating ecological functions of iron tailings, providing a sustainable strategy for tailings ecological restoration and resource utilization.

## Introduction

Iron tailings, the primary solid waste generated from mineral processing, have accumulated in enormous quantities globally due to the rapid development of the mining industry^1,2^. Characterized by extreme nutrient deficiency, compact physical structure, and low biological activity, iron tailings fail to support plant growth and ecological succession, posing severe environmental risks such as soil erosion and ecosystem degradation^3^. Therefore, the ecological remediation of stockpiled iron tailings is urgently required. Over many years, a variety of ecological remediation and utilization methods for tailings have been developed, primarily involving soil replacement and soilification^4–6^. Currently, strategies relying on amendments to enhance nutrient availability are recognized as a more sustainable and effective approach to upgrading barren tailing substrates by improving the extreme conditions of iron tailings and endowing them with ecological functions^7,8^.

In the transition of iron tailings into a soil-like growth medium, the enhancement of ecological functions, community activity, and carbon metabolism are critical prerequisites to the improvement of soil fertility, vegetation colonization, and benign development of the tailings soil. In the previous microcosm study by experts on tailings amended with organic matter, the addition of alfalfa hay or glucose resulted in carbon content enrichment, and consequent increase in available carbon and enhancement of microbial respiration rate^9,10^. These were attributed to the improved physicochemical properties of the tailings by the supplemented organic matter, which in turn improved the living environment of microorganisms and accelerated the soilification of the tailings.

The addition of organic matter was found to enrich indigenous microbial communities by acting as an effective carbon source for functional microbial community in barren soils and deserts^7,8^. It is also noted that a variety of microorganisms survive in the tailings under long-term stockpiling and exposed conditions, which accelerate the carbon metabolism, ecological functions, and soilification process of the tailings. As a result, the addition of organic matter to nutrient-poor tailings would be conducive to the weathering of tailings, as well as accelerate the improvement of tailings microenvironment and the formation of soil-like growth medium, through optimizing indigenous functional microbial community, enhancing their activity and accelerating their carbon transformation function. These speculations gave rise to the present study to investigate whether the addition of exogenous organic matter could affect the indigenous microbial community structure in iron tailings, and thereby change the carbon metabolic pathways of the tailings.

Broadly speaking, microorganisms can be grouped into three groups, based on their carbon metabolic pathways and functions: (a) autotrophic bacteria (e.g., *Thiobacillus spp*); (b) heterotrophic bacteria (e.g., *Rhodobacter*); (c) heterotrophic fungi (e.g., *Trichoderma*), which cannot utilize inorganic carbon and must rely on existing organic carbon as their carbon source and energy source, with the core function of organic carbon decomposition and transformation^11,12^. In many nutrient-poor environments such as deserts, fungi play an important role in the process of carbon metabolism^8^. These fungi may degrade cellulose, lignin, starch, and diverse recalcitrant organic substrates (e.g., plant residues, humus) through secreting extracellular enzymes or forming mycelial networks, under a wide range of stress conditions, ranging from alkaline, carbon-poor to nutrient-leaching conditions^13,14^. Some fungi can survive and drive carbon cycling in harsh environments, including heavy metal stress (e.g., lead-zinc tailings), drought (e.g., desert soil), or low-temperature conditions (e.g., alpine permafrost)^15–17^. Previous mining investigations have revealed that long-term weathered tailings harbor a simple fungal community structure, which is primarily attributed to their harsh physicochemical conditions and nutrient deficiency. We suppose that the exogenous organic matter amendment could stimulate the activity of some functional fungi in the tailings. As one of the objectives, the present study is to characterize the shift of fungal community structure in the tailings after the addition of organic matter.

Humic substances and agricultural waste-derived organic amendments are important for initial eco-engineered tailings-soil formation, as organic carbon is essential to driving microbial carbon metabolism (e.g., optimizing community composition and secreting metabolic enzymes) and enhancing early carbon accumulation in tailings^18,19^. The application of organic amendments is required to optimize the microenvironmental conditions of iron ore tailings^20–23^. However, it is unclear how the supply of different types of organic matter, including easily decomposable and recalcitrant fractions, regulates the responses and interactions among microbial communities, tailings multifunctionality, and organic carbon decomposition potential. It is expected that humic substances and agricultural waste-derived organic amendments would alter the abundance of carbon-metabolizing functional microorganisms by improving tailings physicochemical properties and enhancing tailings multifunctionality.

Therefore, the present study aimed to characterize the changes in tailings multifunctionality and organic matter decomposition potential in response to humic substance and agricultural waste organic amendments, and to reveal their synergistic roles in regulating microbial community composition and carbon metabolism in iron ore tailings. Key questions to be answered include the following: (1) What are the variations in functional diversity and organic matter decomposition potential of iron tailings induced by humic substance and agricultural waste amendments? (2) Can the improvement of tailings microenvironment enhance the abundance of carbon-metabolizing functional microorganisms and the expression of carbon metabolism-related genes? It is hypothesized that (a) organic matter amendment would improve the physicochemical properties of tailings, thereby increasing the tailings multifunctional index and organic carbon decomposition potential; (b) organic matter addition would drive the structural shift of bacterial and fungal communities in tailings;

(c) the physicochemical characteristics and community structure of tailings would affect the relative abundance of functional genes involved in carbon metabolism. Fulvic acid and rice husks were used to amend nutrient-deficient tailings in southern Shaanxi, and investigated the effects of these amendments on the functional indices, microbial communities, and carbon metabolic pathways of iron tailings.

## Materials and methods

### Materials

**Fig. 1:**
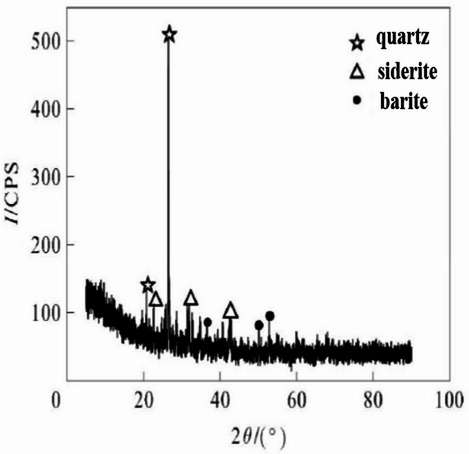
XRD pattern of iron tailings.

Iron tailings were collected in January 2025 from Zhashui County (E 109°10′, N 33°50′), Shaanxi Province, China. The physicochemical properties (Fig. 1; Table 1) of iron tailings were as follows: pH of 7.4 ± 0.05, SiO_2_ content of 45.22%, TFe content of 14.17% and Al_2_O_3_ content of 11.52%. These iron tailings contained no detectable organic matter or heavy metals. The rice husks (RC) used in the experiment were purchased from a rice paddy base in northeast China, and the fulvic acid (FA) was analytical grade with a purity exceeding 98%.

**Table 1:** Chemical analysis for iron tailing.

Component	BaSO_4_	TFe	mFe	SiO_2_	Al_2_O_3_	MgO	K_2_O	CaO	*P*_2_O_5_	Au	Ag	Cu	S	others
% (m/m)	12.31	14.17	0.72	45.22	11.52	3.21	3.87	1.15	0.08	6.10 × 10^− 5^	2.32 × 10^− 4^	0.09	3.51	2.49

### Experimental design

The tailings, after grinding and sieving, were subjected to the subsequent amendment experiments. The selection of organic amendments and experimental design in this study was based on the previous research of Yi et al.^9^. The five experimental treatments were specifically designed as follows: (1) control group (CK): 100 g iron tailings; (2) amelioration with water (WT): 100 g iron tailings + 18 mL water; (3) amelioration with fulvic acid (FL): 100 g iron tailings + 2 g fulvic acid; (4) amelioration with rice husks (RC): 100 g iron tailings + 2 g rice husks; (5) amelioration with fulvic acid and rice husks (FLRC): 100 g iron tailings + 1 g fulvic acid + 1 g rice husks. The tailings amendment experiment was conducted over 60 days, with tailings samples collected on days 1, 7, 15, 45, and 60 to analyze physicochemical properties, functional group composition, element speciation, and biological characteristics.

### Tailings physicochemical analysis

The NH_4_^+^-N, organic matter, available phosphorus, and available potassium in the tailings were determined using Nessler’s reagent colorimetric method^24^, potassium dichromate volumetric method^25^, molybdenum-antimony anti-colorimetric method^26^, and ammonium acetate extraction-flame photometric method^27^, respectively. The pH and salinity of the tailings were measured using a pH meter and a conductivity-salinity meter, respectively.

### Evaluation of multifunctional indices of tailings

We referred to studies on the relationship between soil physicochemical properties and ecological functions, where ecological function diversity was described using a multifunctional index^28–30^. Variables including soil fertility (organic matter), nutrients (NH_4_^+^-N, available phosphorus, available potassium), and salinity-alkalinity (pH, salinity) were used to construct the multifunctional index (MI). The physicochemical parameters were converted to dimensionless form using the following formulas:

1 $$\:\stackrel{\mathrm{-}}{\mathrm{X}}\mathrm{=}\frac{\mathrm{1}}{\mathrm{n}}\sum\:_{\mathrm{i}\mathrm{=1}}^{\mathrm{n}}\mathrm{xi}$$

2 $${\rm \bar{x}' = X\:/X}$$

3 $${\mathrm{x}''= {\rm \bar X} \mathrm{/X}}$$

4 $$\:\mathrm{y'}\mathrm{=}\sum\:_{\mathrm{i}\mathrm{=1}}^{\mathrm{m}}\mathrm{xi}$$

5 $$\:\mathrm{M}\mathrm{I}={\prod\:}_{\mathrm{i}\mathrm{=1}}^{\mathrm{3}}\mathrm{yi}$$

where $\:\stackrel{\mathrm{-}}{\mathrm{X}}$ represents the mean values of each physicochemical parameter, x’ are the dimensionless values of organic matter (OM), NH_4_^+^-N, available phosphorus (AP) and available potassium (AK), x’’ are the dimensionless values of pH and salinity, $\:\mathrm{y'}$ is the comprehensive index of fertility and nutrients, MI is the functional diversity index of tailings.

The change in the multifunctional index of tailings with incubation time was fitted to pseudo-first-order kinetics (*MI*_*t*_=*MI*_*e*_ (1-e^− k*t*^) and pseudo-second-order kinetics(*MI*_*t*_ = *MI*_*e*_^2^k_2_*t*/(1 + *MI*_*e*_k_2_*t*))^28,29^. Here, *MI*_*t*_ and *MI*_*e*_ represent the multifunctional index of tailings at time t and equilibrium, respectively; k and k_1_ denote the pseudo-first-order and pseudo-second-order kinetic rate constants, respectively.

### XPS analysis

The chemical composition and relative contents of C, N and O in tailings samples were analyzed semi-quantitatively via X-ray photoelectron spectroscopy (XPS, Thermo Scientific K-Alpha). First, a survey scan was conducted to identify the elemental types in the samples and their preliminary distribution characteristics. Subsequently, high-resolution scans were performed focusing on the characteristic peak regions of C 1s (280 ~ 295 eV), N 1s (394 ~ 405 eV), and O 1s (525 ~ 540 eV) to accurately obtain data on the chemical states of the target elements and their peak area.

### FTIR spectra

The compositional characteristics of organic matter in tailings were determined by Fourier Transform Infrared Spectroscopy (FTIR). OMNIC software was used to perform spectral baseline correction and calculate the areas of characteristic peaks at 1420, 1536, 1630, 2850, and 2920 cm^− 1^. The organic matter decomposition index (OMDI) was calculated using the following formula:

6 $$\:\mathrm{O}\mathrm{M}\mathrm{D}\mathrm{I}\mathrm{=}\frac{\text{}\mathrm{rA}\mathrm{2850+}\mathrm{rA}\mathrm{2920}}{\mathrm{rA}\mathrm{1630}}$$

where rA2850 and rA2920 represent the characteristic peak areas of aliphatic organic matter, rA1630 represents the characteristic peak area of aromatic organic matter.

### 16 S rRNA gene sequencing and gene functional annotation

Genomic DNA was extracted from 0.5 g tailings samples following the manufacturer’s protocols of the Soil DNA Kit (Shanghai, China). For amplification of the V4-V5 regions of the bacterial 16S rRNA gene, PCR amplification was performed with the universal primer pair 515F (5’-GTGCCAGCMGCCGCGGTAA-3’) and 806R (5’-GGACTACHVGGGTWTCTAAT-3’) to generate raw sequencing data. After data quality control, taxonomic classification information of the bacterial community was obtained. Functional gene and metabolic pathway were predicted via KEGG database and PICRUSt2 (v2.5.0) software. Similarly, fungal communities were analyzed using PCR amplification with the ITS1F/ITS2R primer pair, with the primer sequences listed below: ITS1F: 5’-CTTGGTCATTTAGAGGAAGTAA-3’ and ITS2R: 5’-GCTGCGTTCTTCATCGATGC-3’. This study generated the original sequencing data submitted to the NCBI website *(*https://www.ncbi.nlm.nih.gov/sra/?term=PRJNA1428724*)*, accession number for PRJNA1428724.

### Statistical analysis

Differences among experimental treatments were analyzed using one-way analysis of variance (ANOVA).The correlation between microbial communities and tailings properties was analyzed using Pearson correlation coefficients implemented via the vegan package in R software.

## Results and discussion

### Effect of amendments on the multifunctional indices of tailings

Nutrient-poor tailings environments represent a vital factor limiting functional diversity. To understand the improvement of tailings microenvironment by the addition of amendments, we analyzed samples during the microcosm experiment to examine the physicochemical properties and multifunctional index of the tailings.

#### Physicochemical properties

**Fig. 2:**
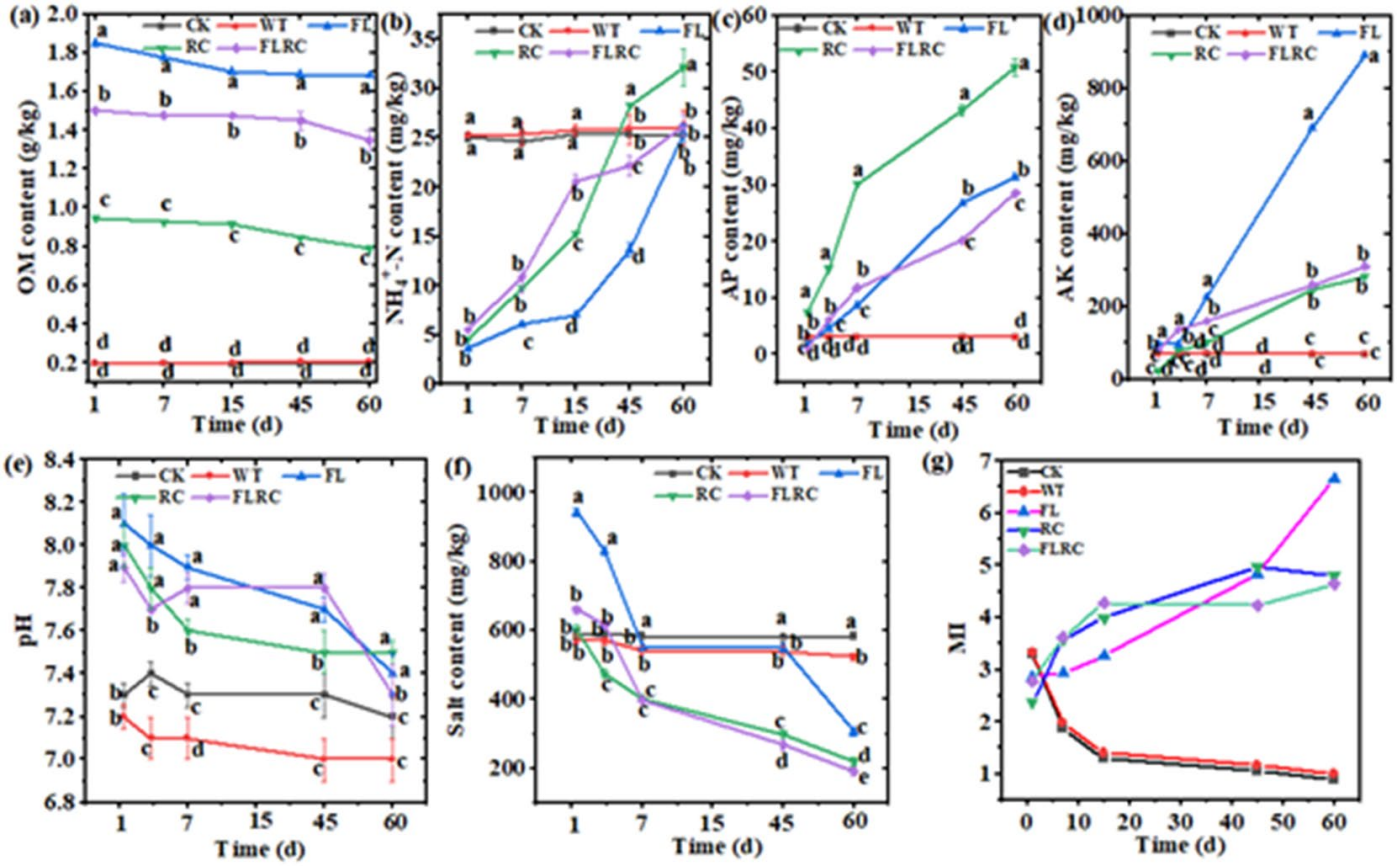
Effects of different amendments on the physicochemical properties of tailings. Different lowercase letters represent significant differences in different treatments at the same time. (a, b, c, d, e, f, and g represent the changes in organic matter (OM), NH_4_^+^-N, available phosphorus (AP), available potassium (AK), pH, salt content, and multifunctional index (MI) with incubation time, respectively).

The overall physicochemical properties of tailings are shown in Fig. 2a and g.

In alignment with our expectations, after 60 days of incubation, in general, there was no significant difference in the physicochemical properties of tailings between the water supplement (WT) and the control treatment (CK). It was mainly attributed to the low clay mineral content in iron tailings, which resulted in weak water absorption capacity^30–32^. The addition of fulvic acid (FL) increased the organic matter, available phosphorus (AP), and available potassium (AK) contents in the tailings by 8.2-, 10.25-, and 12.89-fold compared to the CK, respectively (Fig. 2a, c and d). Treatments with rice husk addition (RC, FLRC) had similar effects as the FL.

Specifically, the RC treatment significantly increased the AP content of tailings to 50.809 mg/kg (Fig. 2c). All treatments (WT, FL, RC, and FLRC) slightly increased the tailings’ pH and significantly reduced their salt content to 190–307.5 mg/kg (Fig. 2e and f), which was because, in the alkaline tailings environment, fulvic acid underwent complexation reactions, while weakly alkaline rice husks exerted an acid-inhibiting effect^33,34^. Yi et al.^9^ used lucerne hay to amend tailings, achieving an increase in the content of TOC in tailings’ pore water from 20 mg/L to 950 mg/L during an 84-day incubation period, which represented a 45-fold increase. Wu et al.^10^ employed root-derived organic matter of spring wheat to remediate fresh iron tailings for 12 months. During this period, the pH showed no significant change, while hot water-extractable organic carbon (HWEOC), salt-extractable organic carbon (SEOC) and TOC increased by more than 2-, 4- and over 4-folds respectively. For glucose mediated remediation, pH slightly decreased; HWEOC, SEOC and TOC increased by 4-, 8- and 7-folds respectively. In the present study, compared with the RC and FLRC treatments, the tailings organic matter content in the FL treatment was 24.85% and 113.31% higher, respectively (*p* < 0.001). The lower organic matter content of tailings in RC and FLRC treatments may be due to the relatively slow decomposition rate of rice husk, which limits microbial accessibility to carbon sources and thus results in a slow increase in carbon reserves over a short period^35,36^. In contrast, direct water supplementation still led to a lack of carbon sources, resulting in no significant difference in tailings organic matter content compared with the control.

#### Multifunctional indices

The multifunctional index (MI) is a critical indicator of microecosystem function, reflecting the potential of biological activity, nutrient metabolism, and energy regulation in tailings. This study examined the effects of external amendments on the multifunctionality index of iron tailings (Fig. 2g). In the control group (CK), MI remained nearly constant at a value of 1. Based on the physicochemical properties of tailings, it can be inferred that the 18% moisture treatment (WT) improved the functional diversity of tailings mainly by influencing NH_4_^+^-N, pH, and salt content within the first 15 days. In the FL treatment, MI increased continuously, which indicated that fulvic acid, as a small-molecule carbon source rich in active functional groups, can sustain tailings’ functions over time. In the RC and FLRC treatments, MI increased rapidly in the first 15 days, followed by a slow increase, reaching values of 4.8 and 4.65, respectively, on day 60. This may be due to the complex carbon components of rice husks, which restricts the multifunctionality of the tailings.

**Fig. 3:**
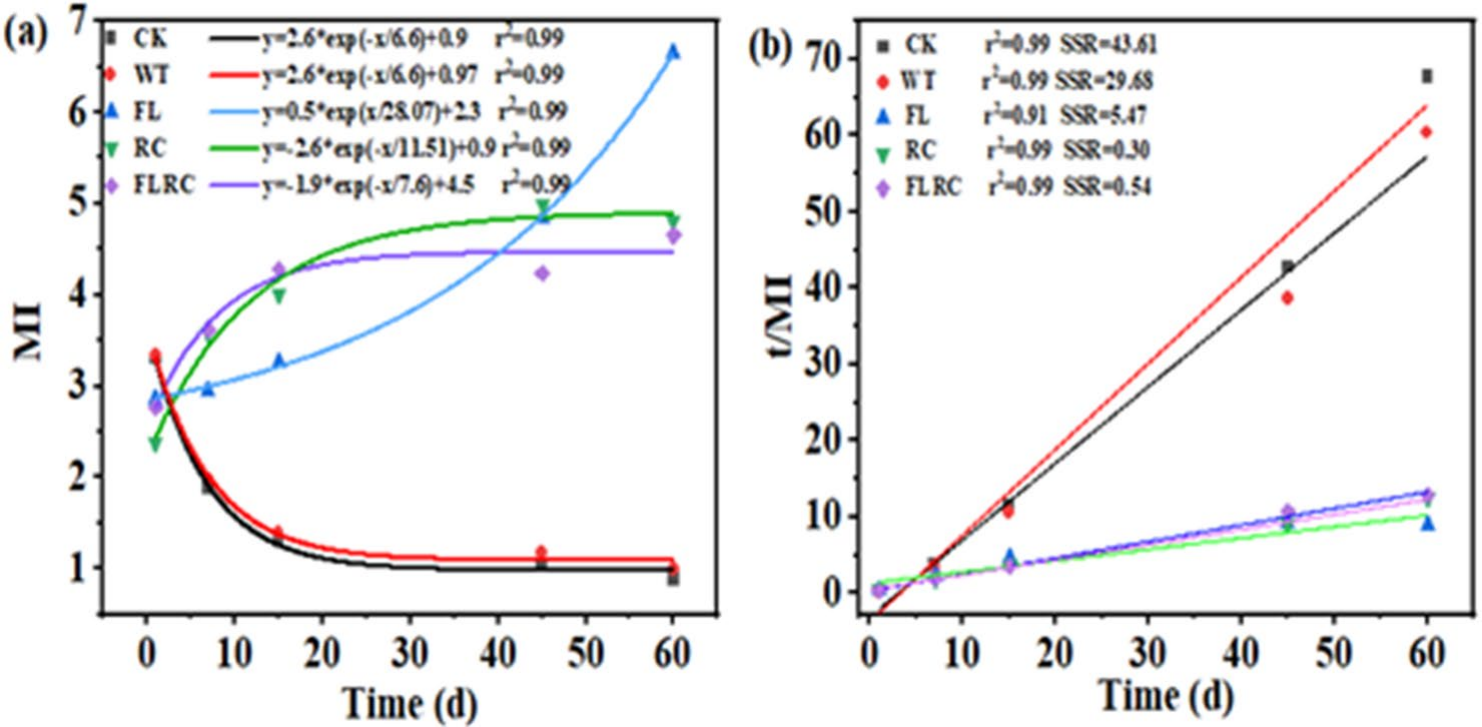
Pseudo-first-order (**a**) and pseudo-second-order (**b**) kinetic equations describing temporal changes in the multifunctional index (MI) of iron tailings.

Pseudo-first-order and pseudo-second-order kinetic models were employed to analyze the amendment effects of the 18% moisture treatment (WT), rice husk treatment (RC), and rice husk combined with fulvic acid treatment (FLRC) on tailings. The correlation coefficients (R^2^), sum of squared residuals (SSR), and rate constants (k) are shown in Fig. 3. Although R² was relatively high for both the pseudo-first-order (Fig. 3a) and pseudo-second-order kinetics (Fig. 3b) (R² ≥ 0.98), the SSR of the pseudo-second-order kinetics (0.30-43.61) was higher than that of the pseudo-first-order kinetics model (0.006–0.09). Thus, the improvement effect of the tailing microcosm conforms to the pseudo-first-order kinetics model. The functional rate constants were 0.1515, 0.1515, 0.0869 and 0.1316 d^− 1^ for the CK, WT, RC, and FLRC treatments, respectively. The increase of MI with amendment time in the treatment with readily available fulvic acid (FL) could be simulated using an exponential equation.

Iron tailings typically exhibit extreme nutrient deficiency and compact physical structure, supplementing them with specific organic amendments is crucial for the success of ecological restoration^37^. Our experiment indicated that single low molecular weight organic acids can continuously improve tailings functions, while fiber-based biomass exhibited an “effect plateau” during tailings amendment. This result has also been reported in previous studies, as evidenced by a study where spring wheat was applied to fresh and aged iron tailings, and the enhancement of biological activity in tailing peaked on day 21 of amendment^10^. In our study, the presence of fiber-based biomass resulted in a limited increase in tailing MI, regardless of whether low-molecular-weight organic acids were added.

In the RC and FLRC treatments, tailings MI exhibited an initial rapid increase followed by a slower rise, MI in the FLRC treatment (2.77–4.28) was higher than that in the RC treatment (2.38-4.00) during 0–15 d, and after 15 d, MI in the RC treatment (4.80–4.97) was higher than that in the FLRC treatment (4.23–4.65) (Fig. 2g). This trend may be attributed to microorganisms preferentially utilizing fulvic acid over rice husks for their rapid stimulatory effect on the tailings microcosm. This preferential utilization results in fulvic acid dominating among available carbon sources in the short term. In addition, the formation of aggregates by fulvic acid and rice husks may lead to an attenuated promotional effect on MI^9^.

In soil systems, excessive organic matter supplementation results in the relative deficiency of nitrogen sources, which in turn causes significant decline in soil quality^38^. In this study, MI was applied to evaluate the functional effects of organic amendments, which was consistent with the soil research findings of a compromise of soil functions with the addition of multiple types of carbon sources. Kiem et al.^39^ reported that the complexity of organic carbon molecular structure is the primary determinant of nutrient utilization efficiency. Interestingly, an 18–25% increase in aromatic carbon content in soil organic matter, there was no significant improvement in soil functional diversity related to biological activity.

### Effects of amendments on the chemical properties of tailings

#### Elemental composition-XPS analysis

**Fig. 4:**
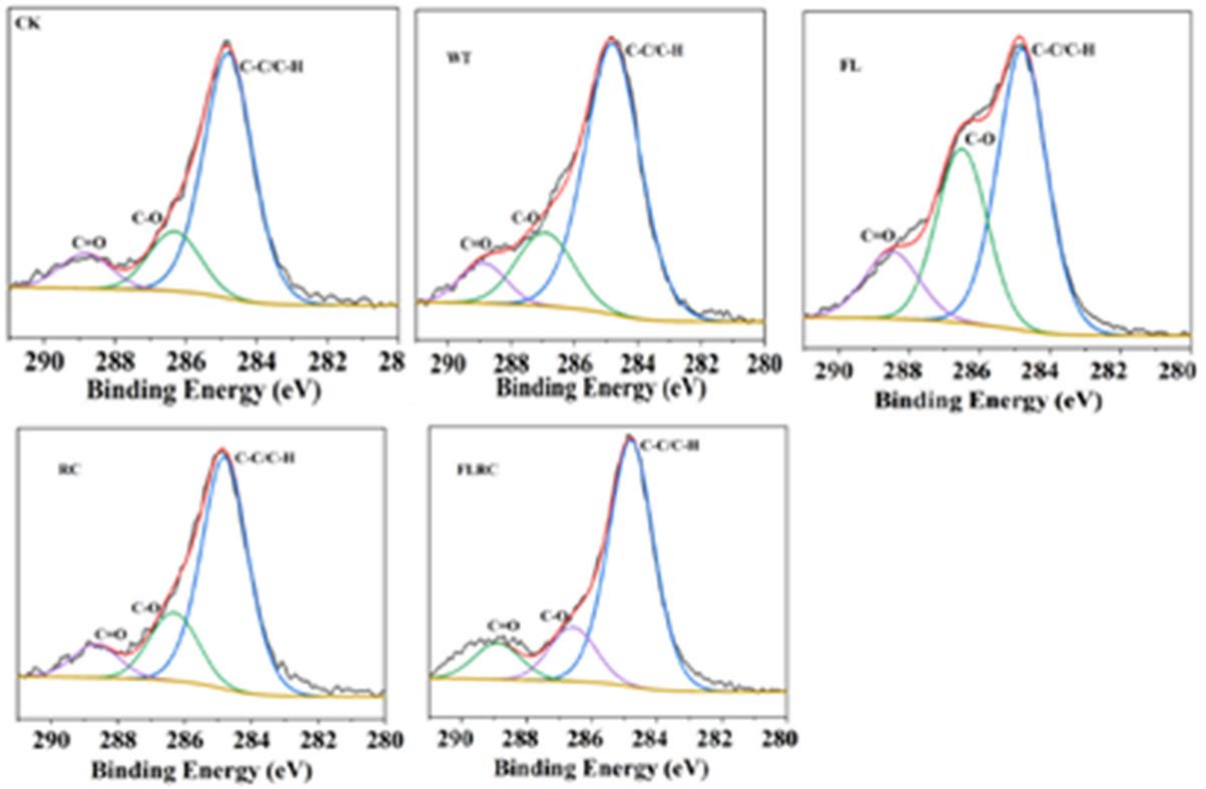
The C1s spectrum of iron tailings after 60 days of incubation.

**Fig. 5:**
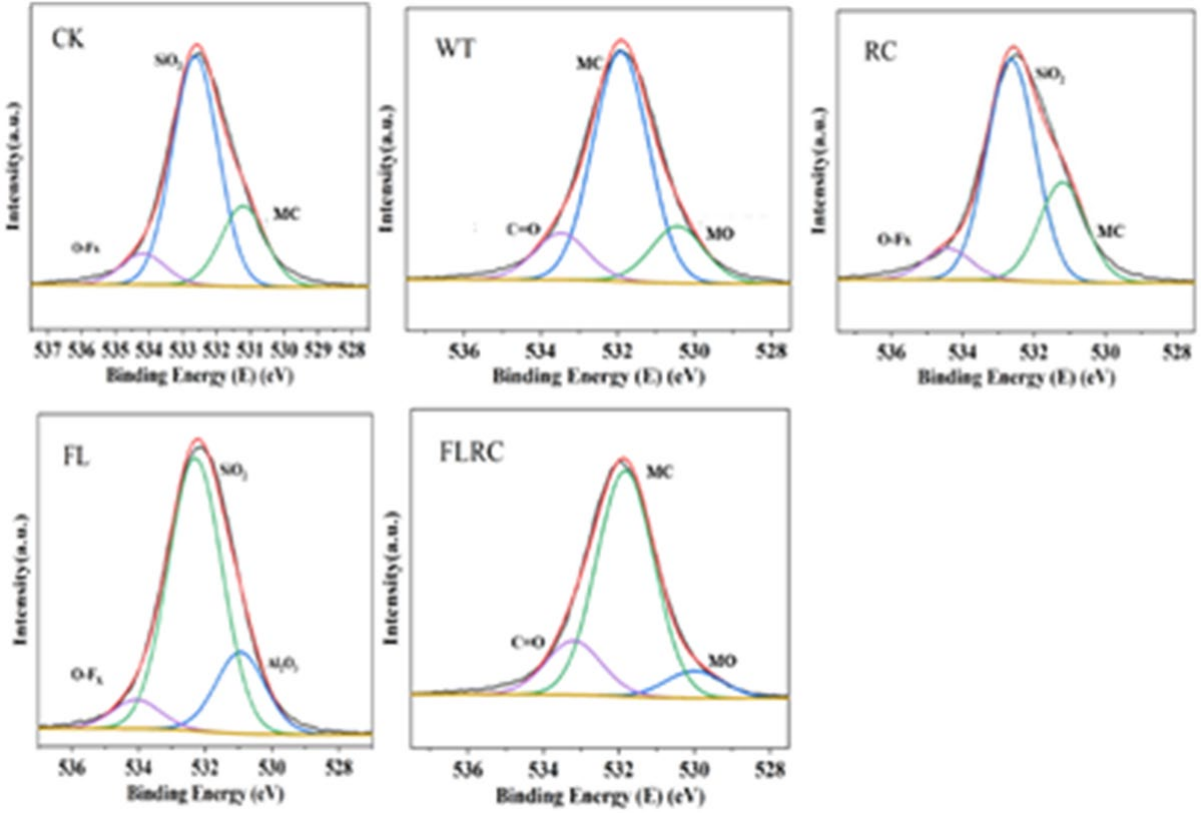
The O1s spectrum of iron tailings after 60 days of incubation.

**Fig. 6:**
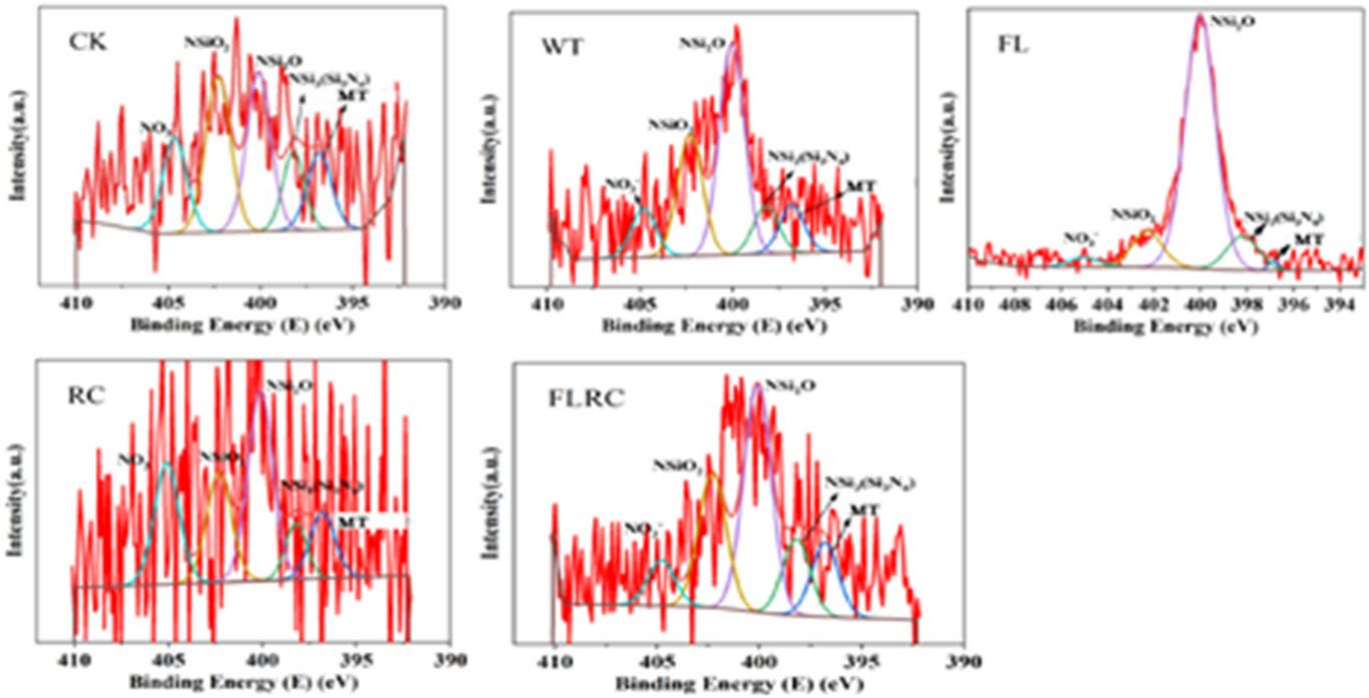
The N1s spectrum of iron tailings after 60 days of incubation.

The changes in C, N, and O contents in tailings were clarified by means of XPS analysis, which covered the survey spectra and high-resolution spectra of C1s, O1s, and N1s (Figs. 4, 5 and 6).

**Fig. 7:**
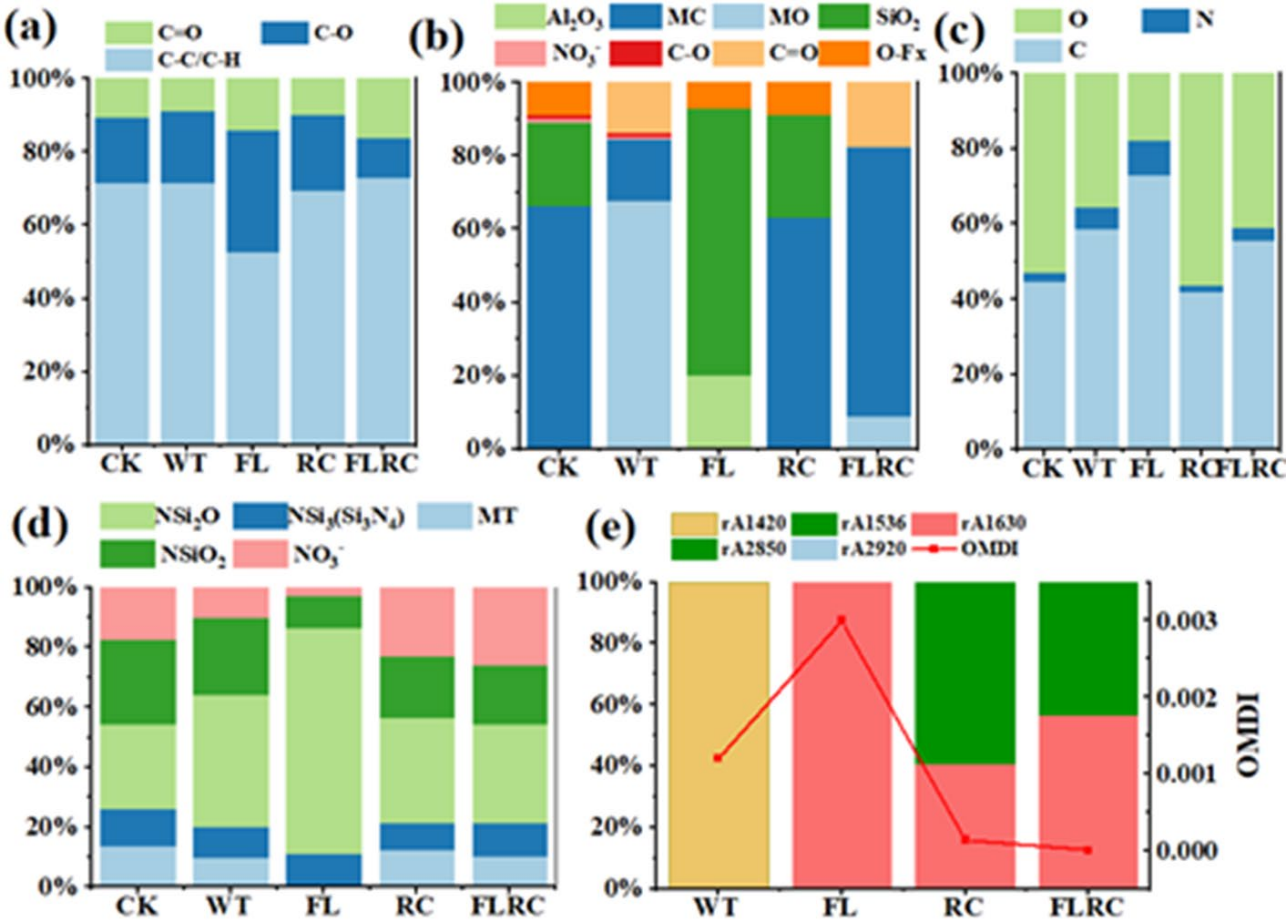
Effects of amendments on the chemical properties of tailings (XPS spectra.

The C-C/C-H bonds remained relatively high (71.83%-73.05%) in the CK, WT, RC, and FLRC, indicating that saturated carbon species were dominant in these treatments (Fig. 7a). In the 18% moisture treatment (WT), the atomic percentages of C-O and C = O showed slight fluctuations, which may be due to the slow oxidation or hydrolysis process promoted by moisture^40^. Compared with CK, the atomic% of C-C/C-H decreased by 26.49%, while the atomic percentages of C-O and C = O increased by 86.27% and 35.5% respectively in the FL. This indicated that fulvic acid provided abundant oxygen-containing functional groups to the tailings, which was the main reason for the continuous increase in tailings’ MI. Previous literature has reported that oxygen-containing functional groups are beneficial for enhancing the nutrient adsorption capacity of tailings, thereby reducing nutrient loss^41^. The atomic% of C-C/C-H, C-O and C = O in RC were comparable to those in FL. Compared with FL and RC, in FLRC, the atomic% of C-O decreased by 47.9–67.5%, while that of C = O increased by 14.8–65.3%. This suggested that fulvic acid and rice husk may have undergone esterification or polymerization reactions^42–44^, converting part of the C-O into C = O, which was also the reason why the MI of FLRC was lower than that of RC after 15 days.

The C-C/C-H bonds remained relatively high (71.83%-73.05%) in the CK, WT, RC, and FLRC, indicating that saturated carbon species were dominant in these treatments (Fig. 7a). In the 18% moisture treatment (WT), the atomic percentages of C-O and C = O showed slight fluctuations, which may be due to the slow oxidation or hydrolysis process promoted by moisture^40^. Compared with CK, the atomic% of C-C/C-H decreased by 26.49%, while the atomic percentages of C-O and C = O increased by 86.27% and 35.5% respectively in the FL. This indicated that fulvic acid provided abundant oxygen-containing functional groups to the tailings, which was the main reason for the continuous increase in tailings’ MI. Previous literature has reported that oxygen-containing functional groups are beneficial for enhancing the nutrient adsorption capacity of tailings, thereby reducing nutrient loss^41^. The atomic% of C-C/C-H, C-O and C = O in RC were comparable to those in FL. Compared with FL and RC, in FLRC, the atomic% of C-O decreased by 47.9–67.5%, while that of C = O increased by 14.8–65.3%. This suggested that fulvic acid and rice husk may have undergone esterification or polymerization reactions^42–44^, converting part of the C-O into C = O, which was also the reason why the MI of FLRC was lower than that of RC after 15 days.

of C1s (**a**), O1s (**b**), survey spectrum (**c**), N1s (**d**), and FTIR spectrum (**e**)).

The original mineral composition of the iron tailings was mainly composed of metal carbonates (67.6%), SiO_2_ (23.37%), and O-containing Fx (9.03%) (Fig. 7b). In the WT treatment, the tailings contained metal oxides (69.32%) and organic C = O (13.84%), indicating that moisture induced carbonate decomposition and metal oxidation (Fig. 7b). After the addition of fulvic acid, the tailings were dominated by SiO_2_ (72.82%) and Al_2_O_3_ (20.13%), implying that fulvic acid primarily targeted the fractions of aluminum and silicon oxides and altered the oxide composition of the iron tailings. The mineral components in the RC were similar to those in the CK. Compared with the FL and RC treatments, the FLRC treatment showed an increase in the atomic percentages of metal carbonates and organic C = O groups, the absence of detectable Al_2_O_3_ and O-containing Fx, and the emergence of metal oxides. This indicated that the combined addition of fulvic acid and rice husk facilitated the enrichment of metal carbonates and the formation of organic carbonyl groups.

The relatively weak and overlapping nitrogen peak in the sample spectra (except for FL) indicated low nitrogen content in the tailings (Fig. 6). The peak positions of each nitrogen-containing component were consistent across different samples, while only the atomic% changed (Fig. 7d). The atomic% of metal nitrides was the smallest in the FL treatment, indicating that fulvic acid strongly inhibited the formation of metal nitrides. Compared with the CK, the atomic% of NSi_2_O increased by 56.95%, 165.64%, 25.20%, and 44.79% in the WT, FL, RC and FLRC treatments, respectively; however, those of NSiO_2_ decreased by 10.18%, 62.16%, 27.42%, and 14.59% respectively. Humidity and fulvic acid inhibited the formation of NO_3_^−^ by 40.80% and 83.29%, respectively; while the treatments containing rice husk facilitated the formation of NO_3_^−^-N, resulting in increases of 31.96% and 87.66%, respectively.

Humidity promoted N accumulation, thereby reducing the C/N ratio. Since fulvic acid contains both C and N, their atomic% in FL (32.39%, 4.14%) were the highest, accompanied by the lowest C/N ratio (Fig. 7c; Table 2). The atomic% of C and N in RC (16.39%, 0.75%) slightly differed from those in CK (14.86%, 0.85%), which resulted in a substantial increase in the C/N ratio. The combined addition of fulvic acid and rice husk caused the compositions of C, N, and O as well as the C/N ratio to tend toward an intermediate level, reflecting the mutual restriction and synergistic regulation between the two amendments.

**Table 2:** The peak areas corresponding to each functional group in the tailing sample.

	C		*N*		O		
	Binding energy(eV)	Atomic %	Binding energy(eV)	Atomic %	Binding energy(eV)	Atomic %	C/*N*
CK	284.8	14.86	402.38	0.85	532.44	84.29	17.48
WT	284.8	16.53	399.9	1.66	531.99	81.81	9.96
FL	284.8	32.39	399.8	4.14	531.96	63.47	7.82
RC	284.8	16.39	401.73	0.75	532.36	82.86	21.85
FLRC	284.7	21.22	401.31	1.36	531.76	77.42	15.60

#### Carbon-containing functional groups-FTIR spectra

To investigate the structure of organic matter in iron tailings, FTIR was used to determine the carbon-containing functional groups in the tailings and clarify the formation process and effect of soil organic matter. Changes in organic matter within the tailings were analyzed by examining the functional groups corresponding to the wavenumbers at 1420, 1536, 1630, 2850 and 2920 cm^− 1^ (Table 3).

**Table 3:** The FTIR spectrum of tailings after 60 days of incubation.

Treatment	WT	FL	RC	FLRC
rA2920	0.08			
rA2850		0.27	0.007	
rA1630		69.84	50.91	40.32
rA1536			73.29	30.58
rA1420	65.82			

Figure 7e indicated that aliphatic carbon chains and carboxylates (rA2920 = 0.08), as well as methyl structures (rA1420 = 65.82) were the main organic matter components in the WT treatment. Fulvic acid contains characteristic functional groups, which resulted in relatively prominent peaks for aliphatic carbon chains (rA2850 = 0.27) and polar oxygen-containing functional groups (rA1630 = 69.84) in the tailings samples (Fig. 7e). Nitrogen-containing functional groups (rA1536 = 73.29) were the most dominant in the RC treatment. For the FLRC treatment, rA1630 (40.32) was lower than that in FL (69.84) and RC (50.91), while rA1536 (30.58) was also lower than that in RC (73.29). This indicated that the two amendments (fulvic acid and rice husk) interacted, leading to the weakened signals of these functional groups.

OMDI stands for the proportion of easily decomposable components in organic matter and their decomposition potential^45,46^, with values of 0.0012 (WT), 0.003 (FL), 0.00014 (RC), and 0 (FLRC) respectively (Fig. 7e). This indicated that the addition of fulvic acid was conducive to increasing OMDI and enhancing the decomposition potential of organic matter, while the application of rice husk reduced OMDI and diminished this decomposition potential. In the FLRC treatment, due to the loss of readily decomposable aliphatic components, the organic matter was dominated by its recalcitrant fraction. Thus, the decomposition potential of organic matter was minimized, which was also the reason for the lower MI in FLRC compared to RC after 15 d.

### Effects of amendments on the microbial community structure of tailing

#### Bacterial community

Five tailing samples, CK, WT, FL, RC and FLRC after 60 d of microcosm cultivation were selected for high-throughput sequencing. High-throughput paired-end sequencing of 16 S rRNA gene amplicons from tailings samples generated between 46,418 and 85,323 clean reads per sample, with sequence lengths ranging from 207 to 330 bp and a mean length of approximately 256 bp, indicating that the sequencing quality was sufficient for subsequent bacterial diversity analysis^47–49^. The Ace, Simpson, and Shannon indices were employed to assess the richness and evenness; in the tailings samples, where the Ace and Shannon indices primarily reflect community richness and the Simpson index reflects community evenness^50–52^.

Overall, rice husk was most effective in enhancing the diversity and richness of microbial communities in tailings with the highest Ace (1067) and Shannon indices (4.59) among all treatments (Table 4). Specifically, the Ace index of the 1% fulvic acid treatment (FL) and the Shannon index of the 18% moisture treatment (WT) were lower than those of the other samples, and the Simpson index was relatively high in both WT and FL, indicating that fulvic acid and moisture stimulated the dominance of a small number of adapted microorganisms.

**Table 4:** Diversity indices of bacterial community in tailing samples.

Treatment	shannon	ace	simpson
CK	4.56748	724.62207	0.02946
WT	2.97802	593.40308	0.14997
FL	3.00664	465.27304	0.11234
RC	4.59838	1066.98326	0.0473
FLRC	3.99935	840.04236	0.06242

**Fig. 8:**
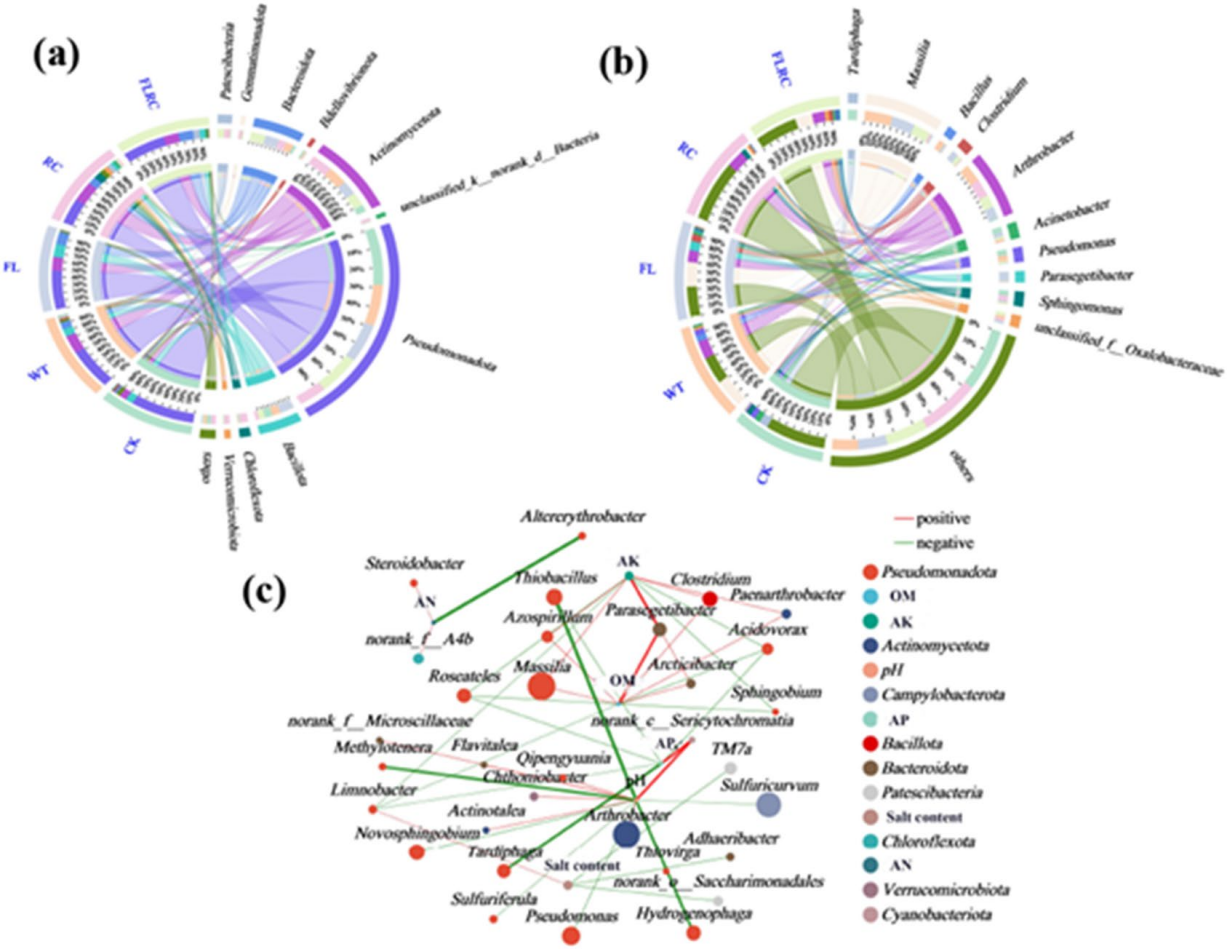
Relative abundances of bacterial communities at the phylum (**a**) and genus (**b**) levels after 60 days of incubation, and correlation analysis (**c**) between tailings physicochemical properties and dominant bacteria genera.

*Pseudomonadota* and *Actinomycetota* were the dominant bacterial phyla across all treatments (Fig. 8a). However, compared with CK, the relative abundance of *Pseudomonadota* decreased by 55.56%, 32.20%, 27.53%, and 32.81% in the WT, FL, RC, and FLRC treatments, respectively; conversely, that of *Actinomycetota* increased by 2.01-, 1.08-, 1.70-, and 1.08-fold in these treatments, respectively. This result indicated that *Pseudomonadota* adapted to the original oxygen-sufficient environment in tailings and was independent of readily available carbon for survival. In contrast, *Actinomycetota* is hygrophilic and capable of utilizing organic matter, and even decomposing recalcitrant carbon. Previous literatures have reported that *Actinomycetota* can decompose complex organic substances in tailings via various enzymes thereby enhancing nutrient availability^53–55^.

At the genus level, in general, a significant change in bacterial community structure was detected between the original (CK) and all treated tailings (Fig. 8b). Specifically, *Tardiphaga* (10.35%), *Acinetobacter* (6.50%), *Pseudomonas* (6.12%), *and Sphingomonas* (5.73%) were the dominant genera in CK. The 18% moisture treatment (WT) promoted *Arthrobacter* to become the most dominant genus, with a relative abundance of 19.27%. After the addition of organic matter (OM) amendments, *Massilia* and *Arthrobacter* emerged as the dominant genera across the FL, RC, and FLRC treatments. Their relative abundances were 26.60% and 12.93% in FL, 31.96% and 19.66% in RC, and 19.83% and 14.17% in FLRC, respectively. Notably, *Tardiphaga*, a dominant genus in CK, is slow-growing and possesses the potential for nutrient retention in aerobic environments. This indicates that nutrient-poor tailings inherently harbor nutrient-acquiring microbial taxa. Previous literature has reported that *Massilia* and *Arthrobacter* are chemoheterotrophic bacteria and their relative abundances are regulated by organic matter inputs^56^.

A two-factor network correlation analysis was conducted to analyze the correlations between the dominant bacterial genera and the physicochemical properties of the tailings (Fig. 8c). Correlations between pH and various bacterial taxa were observed, which reflected that these microorganisms were sensitive to pH changes in the tailings. In contrast, NH_4_^+^-N was found to exclusively correlate with *Altererythrobacter* and *Steroidobacter*, suggesting only a small number of microorganisms were involved in the NH_4_^+^-N transformation process.

Nodes corresponding to *Massilia*, *Pseudomonas*, and *Arthrobacter* were larger in size, indicating these genera play an important role for sustaining the functional diversity of tailings. Specifically, the correlation coefficients were as follows: 0.9 between *Arthrobacter* and pH, and between *Massilia* and organic matter (or AK); 0.97 between *Sericytochromatia* and pH (or AP), and between *Parasegetibacter* and organic matter (AK). Separately, the genus of *Sulfuricurvum* exhibited a negative correlation with pH. However, the relative abundance of *Arthrobacter*, *Sulfuricurvum*, and *Massilia* were higher than those of *Sericytochromatia* and *Parasegetibacter.* As indicated in Sect.  3.1.1, the addition of organic amendments significantly altered the physicochemical properties of tailings. Therefore, the indigenous microorganisms in tailings exhibited selectivity in the utilization of organic nutrients, and it was bacteria with a relatively low relative abundance (< 1%) rather than dominant ones that immobilized nutrients during the tailings soilization process. Jin et al.^57^ investigated the diversity of soil bacterial and fungal communities during the ecological restoration of mining areas, and determined that the distribution characteristics of fungi and dominant fungal taxa exert a decisive influence on soil functions. Li et al.^58^ analyzed the composition and structure of microbial communities in the process of tailings ecological restoration, and found that it is specific key functional microorganisms rather than dominant microbial groups that drive the diversity of ecological functions in tailings regions. The findings of this study are consistent with these of the previous research.

#### Fungal community

Tailings samples incubated in microcosms for 60 days were followed by high-throughput sequencing to analyze the fungal community structure.

**Fig. 9:**
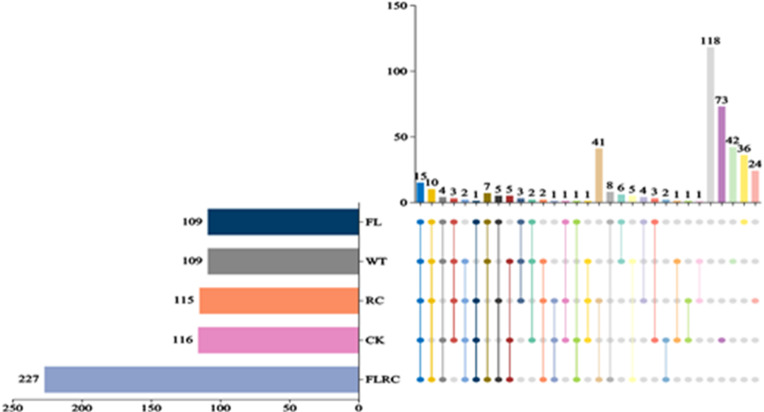
Number of OTUs in the tailings fungal community (left: bar chart of total OTU counts; right: number of unique OTUs).

The number of OTUs showed minimal variation between the CK (116) and RC (115), as well as WT (109) and FL (109), while FLRC reached 227 OTUs (Fig. 9), indicating that the combined amendment of rice husks and fulvic acid significantly enhanced the fungal OTU richness in the tailings. The number of unique OTUs was the highest in FLRC (118), while the number of shared OTUs among the four groups (CK, WT, FL, and RC) was relatively low. This demonstrated that the co-addition of rice husks and fulvic acid not only increased the total number of OTUs, but also effectively enriched the specific fungal taxa in tailings.

The fungal community diversity in the original tailings (CK) was the highest with a Shannon index of 3.43 and a Simpson index of 0.093, yet it lacked distinct dominant species (Table 5). The treatments with single amendments (WT, FL and RC) all reduced fungal species diversity, among these, the RC treatment exhibited the lowest diversity, while the co-addition of organic amendments yielded the most pronounced increase in fungal richness.

**Table 5:** Diversity indices of fungal community in tailing samples.

Treatment	shannon	ace	simpson
CK	3.43451	116.3339	0.09347
WT	2.42563	109.5236	0.2159
FL	2.63974	109.5748	0.12873
RC	1.73814	120.6494	0.3697
FLRC	2.51807	238.984	0.15058

**Fig. 10:**
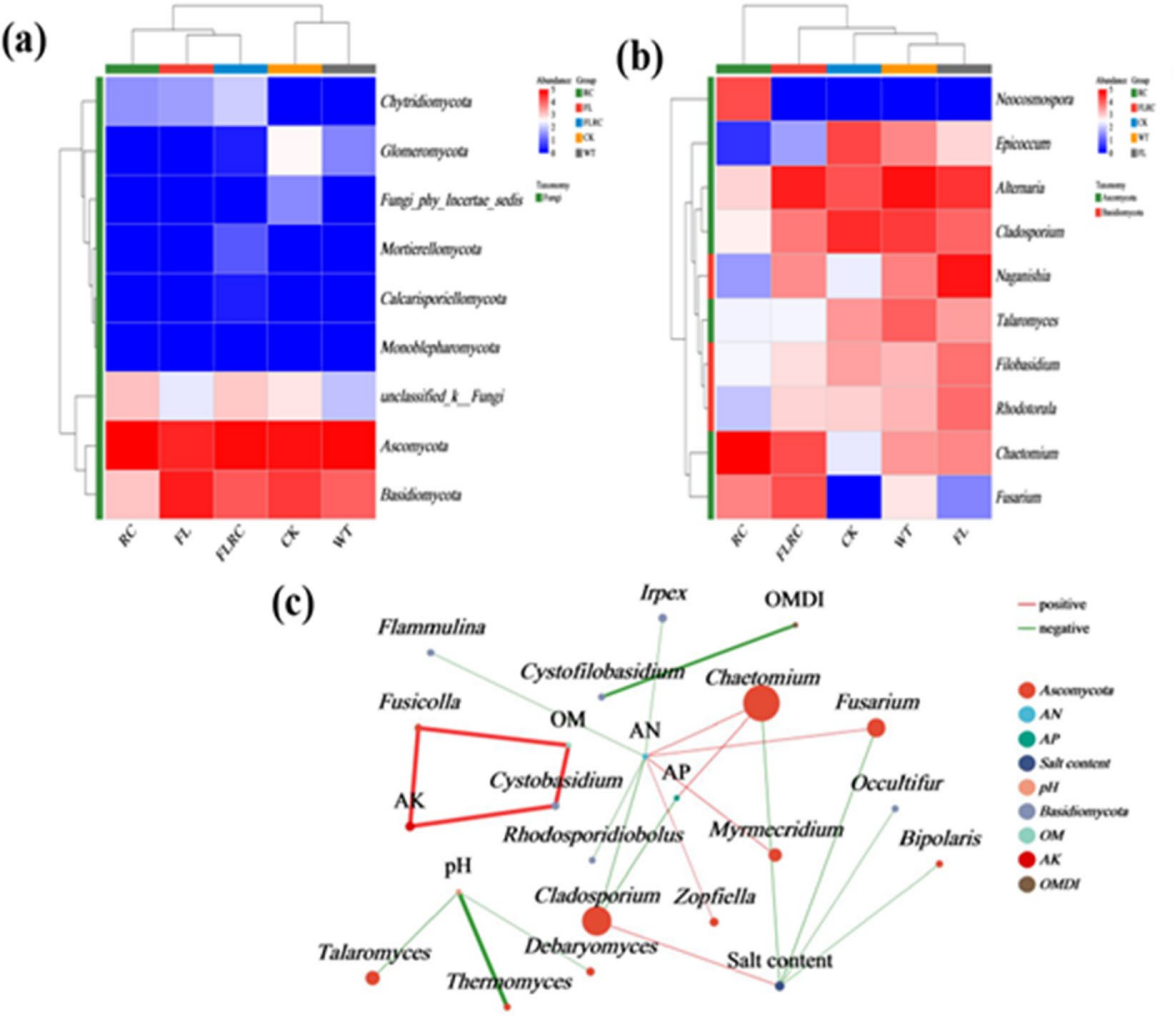
Heatmap of relative abundances of fungal community at the phylum (**a**) and genus (**b**) levels in different treatments (CK, WT, FL, RC and FLRC) and correlation analysis between tailings physicochemical properties and dominant fungal genera.

Hierarchical clustering heatmap analysis was employed to characterize the dominant fungal phyla and genera in tailings (Fig. 10a and b). Overall, the community structure of fungi was simpler than that of bacteria. At the phylum level, *Ascomycota* appeared dark red across all treatments, serving as the core dominant fungal phylum with relative abundances of 69.85%, 87.40%, 44.72%, 96.85%, and 83.47%, respectively (Fig. 10a). *Basidiomycota* was identified as the subdominant phylum. Notably, amendments had a minimal impact on phylum-level fungal communities. Subtle variations were only observed in low-abundance and unclassified fungi. Collectively, *Ascomycota* and *Basidiomycota* accounted for over 99% of the total fungal community. This phenomenon can be attributed to their strong environmental adaptability and efficient resource utilization capabilities^59,60^.

At the genus level, amendments exerted a significant impact on the fungal community structure in the original iron tailings (Fig. 10b). The relative abundances of *Epicoccum* and *Fusarium* were the highest and the lowest respectively, in the CK. It has been reported that *Epicoccum* was a salt-alkali tolerant fungal genus involved in organic matter decomposition, while *Fusarium* was a soil-borne pathogenic fungal genus^61,62^, . After 60 days of amelioration, the relative abundance of *Epicoccum* was the lowest in the RC treatment, while that of *Fusarium* increased steadily across all treatments, indicating the RC was not a preferred carbon source for *Epicoccum*. *Neocosmospora* and *Naganishia* were identified as the dominant genera in the RC and FL, respectively. Notably, rice husks (in the RC and FLRC) facilitated *Chaetomium*’ s emergence as a dominant genus. Overall, different amendments selectively enriched specific dominant fungal genera in the tailings, which exhibited distinct distribution patterns. *Neocosmospora* was recognized as a core fungal genus capable of secreting fructosyltransferase to catalyze fructooligosaccharides biosynthesis, *Naganishia* was closely associated with carbohydrate fermentation, and *Chaetomium* played a vital role in litter decomposition within tailings ecosystems^63–65^.

Two-factor network analysis revealed that *Fusarium* and *Cystobasidium* both showed a positive correlation coefficient of 0.97 with OM and AK (Fig. 10c), which was consistent with the findings of Wang et al. and Milanović et al.^62,66^. Meanwhile, *Chaetomium* displayed significantly positive correlations with NH_4_^+^-N and AP, whereas *Cladosporium* showed a significantly negative correlation coefficient of 0.9 with NH_4_^+^-N and P. The relative abundances of *Fusarium* and *Cystobasidium* (< 0.01) were lower than those of *Chaetomium* (0.002–0.58) and *Cladosporium* (0.004–0.24). Furthermore, OMDI exhibited a negative correlation with *Cystofilobasidium* and *Flammulina*. This observation indicated that, compared with higher-abundance fungi, low-abundance fungi (*Fusarium* and *Cystobasidium*) exhibited a stronger correlation with the physicochemical properties of the iron tailings. Previous studies reported that the relative abundances of *Cladosporium*, *Cystofilobasidium* and *Flammulina* in soil were influenced by organic matter and pH^67–69^.

### Effects of amendments on the carbon transformation function of tailings

#### Bacterial carbon metabolic function

**Fig. 11:**
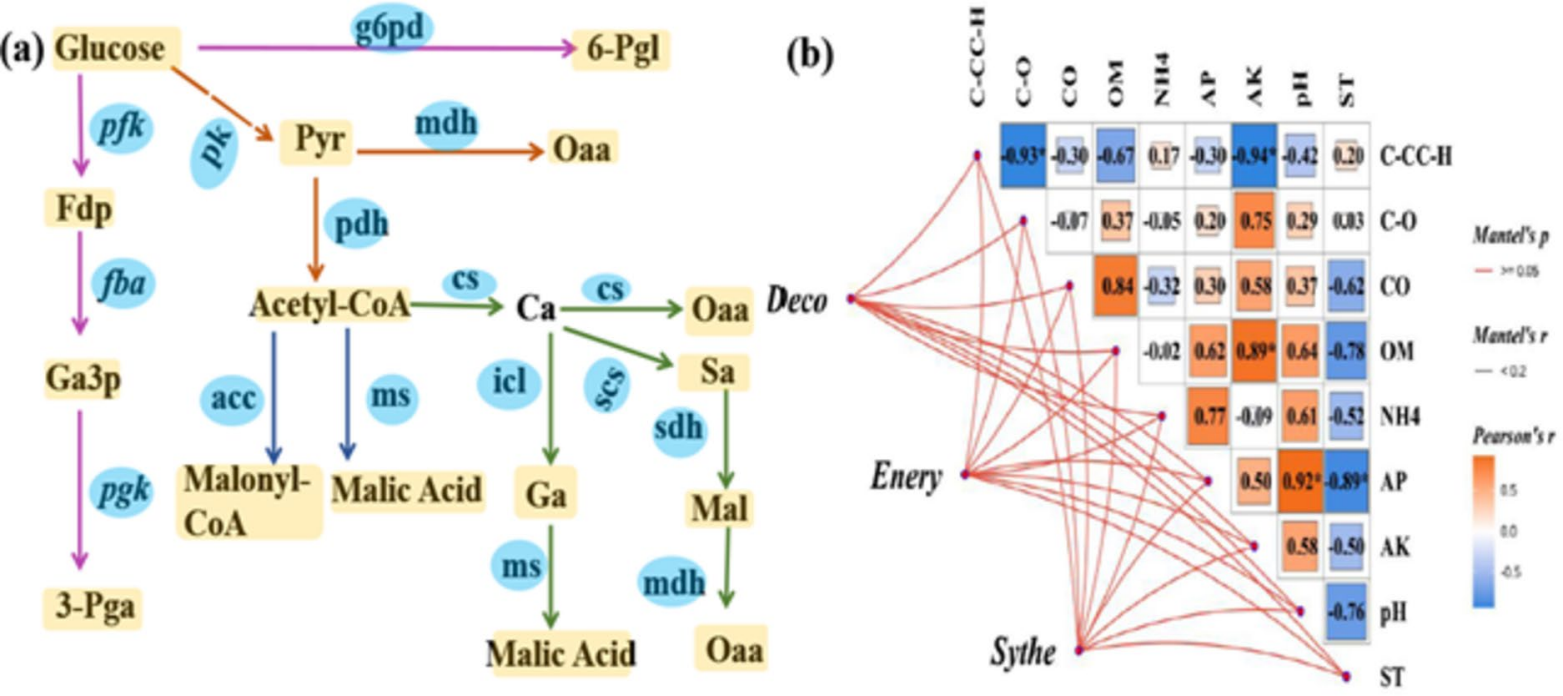
The tailings carbon cycle^61^(**a**) and the correlation between functional gene and tailings properties by Mantel’s test (**b**). Notes: Yellow rectangles represent substances, and blue ellipses represent genes involved in regulating the activity of key enzymes in carbon metabolism pathways. For panel (b): Mantel test was used to analyze the association between bacterial functional gene groups and tailings properties (red line indicates significance at Mantel test *p* ≥ 0.05); the color intensity represents the correlation between tailings properties (orange and blue indicate a positive and negative correlation, respectively). (Abbreviations: AP, available phosphorus; AK: available potassium; OM: organic matter; AN: ammonia nitrogen. Deco, Enery, and Sythe represent the relative abundances of genes encoding enzymes involved in carbon source decomposition, energy production, and synthesis, respectively.)

Carbon metabolism-related genes and pathways in tailings were identified via KEGG analysis (Fig. 11a), and 9 carbon conversion pathways were characterized as follows^70^: (1) Glycolysis: The pathway converts glucose to fructose-1,6-bisphosphate (Fbp) through the catalytic action of phosphofructokinase (*pfk*, EC: 2.7.1.11), then to glyceraldehyde-3-phosphate (Ga3p) by fructose-bisphosphate aldolase (*fba*, EC: 4.1.2.13), and subsequently to 3-phosphoglycerate (3-Pga) by phosphoglycerate kinase (*pgk*, EC: 2.7.2.3); (2) Pentose phosphate pathway: Glucose is converted to 6-phosphogluconolactone (6-Pgl) by glucose-6-phosphate dehydrogenase (*g6pd*, EC: 1.1.1.49); (3) Pyruvate metabolism: Glucose is converted to pyruvate (Pyr) by pyruvate kinase (*pk*, EC: 2.7.1.40), and Pyr is converted to acetyl-coA by pyruvate dehydrogenase (*pdh*, EC: 1.2.4.1); (4) Pyruvate carboxylation: Pyruvate is directly converted to oxaloacetate (Oaa) by malate dehydrogenase (*mdh*, EC: 1.1.1.37); (5) Tricarboxylic acid (TCA) cycle: Acetyl-coA is converted to citrate (Ca) by citrate synthase (*cs*, EC: 2.3.3.1), and Ca is subsequently converted to oxaloacetate (Oaa) via sequential reactions of other enzymes in the TCA cycle; (6) Glyoxylate cycle: Ca is converted to glyoxylate (Ga) by isocitrate lyase (*icl*, EC: 4.1.3.1), and Ga is further converted to malate by malate synthase (*ms*, EC: 2.3.3.9); (7) TCA cycle (succinate-malate branch): Ca is first converted to succinyl-CoA, then to succinate (Sa) by succinyl-CoA synthetase (scs, EC: 6.2.1.5); succinate is further converted to fumarate by succinate dehydrogenase (sdh, EC: 1.3.5.1), and fumarate is finally converted to malate (Mal) by fumarase (fum, EC: 4.2.1.2); malate is then converted to Oaa by mdh; (8) Fatty acid biosynthesis initiation: Acetyl-CoA is converted to malonyl-coA by acetyl-CoA carboxylase (*acc*, EC:6.4.1.2); (9) Glyoxylate cycle (acetyl-CoA-malate branch): Acetyl-CoA is directly converted to malate by malate synthase (*ms*).

When different types of amendments were applied for iron tailings remediation, the abundances of genes encoding key enzymes across various metabolic pathways exhibited substantial changes (Table 6). In the CK, the abundances of almost all genes encoding enzymes were the lowest. The abundances of *pdh*, *pc*, and cellulase in CK were 24530.31, 5376.23, and 17234.97, respectively. In the RC, these values were 2-fold, 2-fold, and 3-fold higher than those in CK, indicating that rice husk addition significantly promoted the conversion of pyruvate to acetyl-coA, cellulose degradation, and the tricarboxylic acid (TCA) cycle.

**Table 6:** The relative abundance of genes that control key enzymes in the carbon cycle.

KO number	KO description	CK	WT	FL	RC	FLRC
K00850	6-phosphofructokinase (pfk) [EC:2.7.1.11]	19410.50	39098.17	33546.18	35928.86	34026.86
K01622	fructose 1,6-bisphosphate (fbp) aldolase/phosphatase [EC:4.1.2.13 3.1.3.11]	7	23	0.008	412	33
K00873	pyruvate kinase (pk) [EC:2.7.1.40]	35632.04	47304.81	38407.11	45793.38	43653.68
K00927	phosphoglycerate kinase (pgk) [EC:2.7.2.3]	34905.36	43892.82	37217.00	48727.47	43786.36
K00161	pyruvate dehydrogenase (pdh) E1 component alpha subunit [EC:1.2.4.1]	24530.31	56524.12	43894.14	60755.83	46712.42
K01958	pyruvate carboxylase (pc) [EC:6.4.1.1]	5376.23	14525.56	8700.20	15678.77	12081.99
K01647	citrate synthase (cs) [EC:2.3.3.1]	49042.92	69962.23	59110.19	56835.45	59052.65
K01902	succinyl-CoA synthetase alpha subunit (scs) [EC:6.2.1.5]	33093.32	43274.84	36523.35	42497.27	39508.94
K00239	succinate dehydrogenase / fumarate reductase, flavoprotein subunit (sdh) [EC:1.3.5.1 1.3.5.4]	38674.77	47389.45	37580.8	44293.88	41171.37
K00036	glucose-6-phosphate 1-dehydrogenase (g6pdh) [EC:1.1.1.49 1.1.1.363]	34568.55	64204.5	46951.48	59689.92	51055.26
K01637	isocitrate lyase (icl) [EC:4.1.3.1]	23356.94	35360.13	26797.19	20880.52	26691.31
K01638	malate synthase (ms) [EC:2.3.3.9]	27118.84	47782.06	33876.38	32613.6	35145.97
K01961	acetyl-CoA carboxylase, biotin carboxylase subunit (acc)[EC:6.4.1.2; 6.3.4.14]	34586.55	45807.09	37831.17	37234.61	37848.9
K01179	endoglucanase (cellulase) [EC:3.2.1.4]	17234.97	26093.02	19625.83	34428.13	25110.97
K00026	Malate dehydrogenase (mdh)[EC: 1.1.1.37]	19410.5	39098.17	33546.18	35928.86	34026.86
	Pyruvate dehydrogenase (acetyl-transferring)	82926.27	154982.15	156623.63	117771.19	125062.56

Notably, the relative abundances of *pfk*, *pk*, *pgk*, *pdh*, *cs*, *scs*, and *sdh* in FL were higher than those in CK but lower than those in RC, indicating that fulvic acid, as a readily available small-molecule carbon source, can rapidly activate energy-generating metabolism and the glyoxylate cycle^71^. The relative abundances of *pfk*, *pk*, *pgk*, *pdh*, *pc*, *cellulase*, *acc*, and *g6pdh* in FLRC were all intermediate between those in FL and RC, indicating that the simultaneous application of fulvic acid and rice husk exerted both synergistic and competitive effects during the carbon cycle^72^. In general, among the five treatments, the vast majority of genes encoding key enzymes in the carbon cycle displayed the highest abundances in the WT, which indicated that increasing humidity comprehensively activated carbon cycling pathways.

Correlation analysis based on the Mantel test revealed that the three gene categories were mostly correlated with soil physicochemical factors and functional groups (Fig. 11b). Genes related to decomposition (*Deco*) displayed the most pronounced correlations with oxygen-containing functional groups (C-O, C = O) and nutrient factors (AP, AK), which reflected that the properties of external carbon sources were the most important factors regulating the carbon cycle in tailings. Genes associated with energy metabolism (*Enery*) and synthesis (*Sythe*) demonstrated strong correlations with OM, pH, and salt content, suggesting that the tailings microenvironment was the key factor directly propelling the carbon cycle.

#### Fungal carbon metabolic function

The abundance of enzymes involved in carbon metabolism varied across tailings treated with different amendments (Table 7).

**Table 7:** The relative abundance of genes encoding key enzymes in the carbon cycle.

Enzyme number	Enzyme category	CK	WT	FL	RC	FLRC
4.1.3.1	Isocitrate lyase (icl)	112,754	113,180	111,827	113228.42	113049.28
1.1.1.49	Glucose-6-phosphate dehydrogenase (NADP(+))	57,419	57,245	57,236	57455.71	57314.14
1.2.4.1	Pyruvate dehydrogenase (acetyl-transferring)	144,993	159,107	137,628	133469.13	148878.42
2.3.1.7	Carnitine O-acetyltransferase	135,947	122,209	148,537	116748.13	125386.42
2.7.1.11	6-phosphofructokinase	58,799	58,234	57,552	57373.71	57340.14

**Fig. 12:**
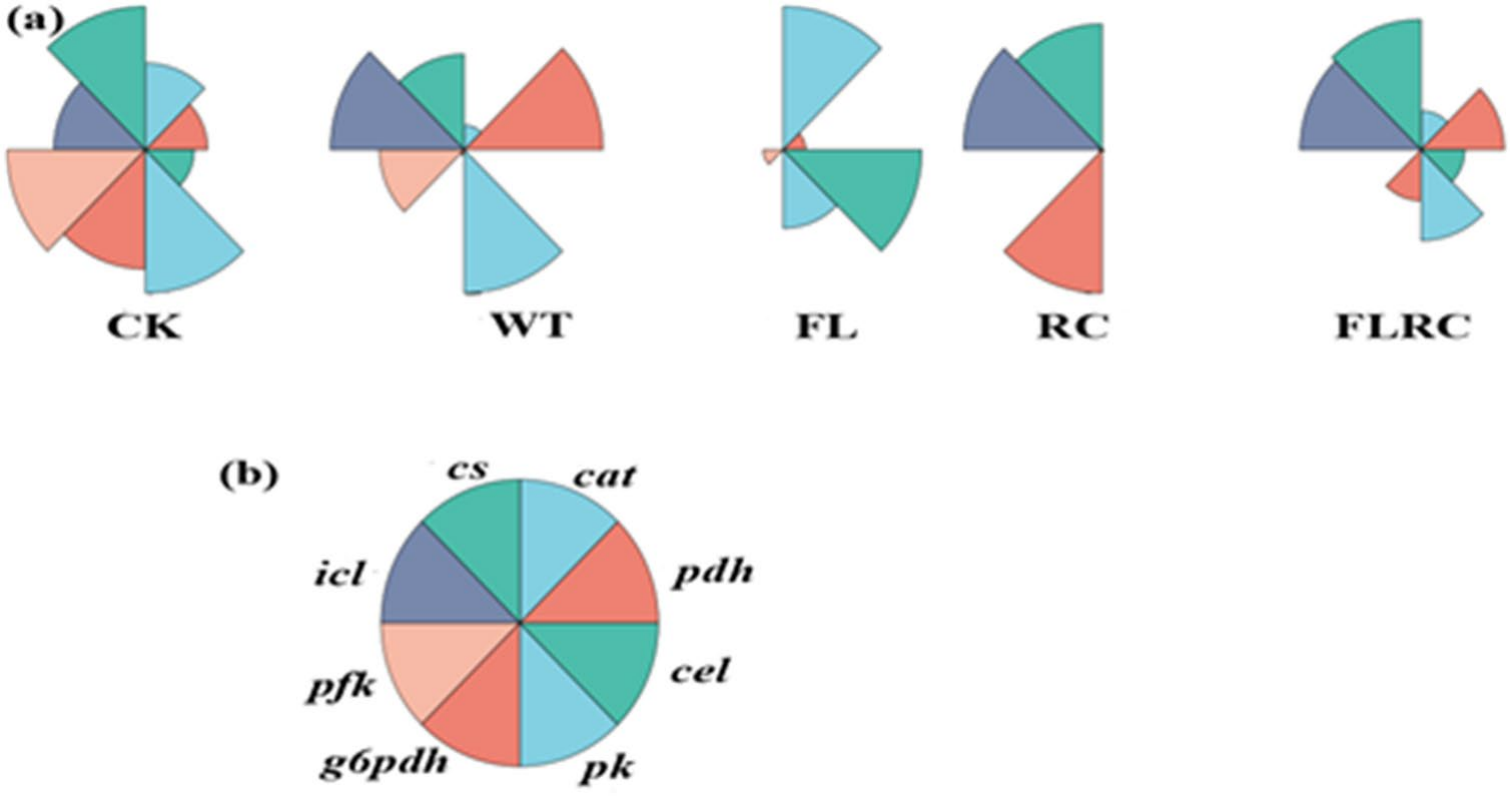
Relative abundance of genes controlling key enzymes in fungal carbon metabolism among different treatments. (a) Comparison of sector distribution of the above genes among treatments (CK, WT, FL, RC, FLRC); (b) Legend for the genes. Note: With identical sector width, the sector area increases as the radius grows, representing an increasing trend in the relative abundance of genes controlling key enzymes.

Enhanced humidity induced only a slight increase in the abundance of *icl* and *pdh* in the WT (113180, 159107) than in the CK (112754, 144993). This was consistent with findings reported in literature, which indicated that appropriate humidity could maintain the osmotic balance of fungal cells to some extent and thereby promote hyphal growth and nutrient uptake^73^. Compared to CK (16206, 135947), fulvic acid enhanced the enzymes abundances of *cel* and *cat* (32372, 148537) in the FL (Fig. 12; Table 6). This may be attributed to the fact that fulvic acid, as a readily available carbon source, directs excess carbon to lipid synthesis after rapidly supplying energy. The abundances of *pdh* and *cat* in RC were the lowest among all samples, which is due to the slow decomposition of complex carbon in rice husks and the resulting insufficient carbon source supply^74^. After the application of organic amendments, the abundances of *cel* and *cat* across samples followed the order RC < FLRC < FL, while that of *icl* followed FL < FLRC < RC. This indicated that, compared to single amendments, the co-application of fulvic acid and rice husk enhances multiple carbon metabolic pathways to a greater extent.

Overall, different types of amendments can effectively enhance specific carbon decomposition and synthesis pathways. The addition of readily available carbon (fulvic acid) stimulated glycolysis and fatty acid synthesis, while the supplementation of recalcitrant carbon (rice husk) promoted the glyoxylate cycle. When readily available and recalcitrant carbon were co-added in combination, these pathways were simultaneously enhanced.

## Conclusions

In this study, two types of carbon sources, fulvic acid and rice husk, were used for the comparative amendment of iron tailings. Owing to differences in oxygen-containing functional groups and organic matter decomposition potential between the two amended tailings, the multifunctional index exhibited exponential growth and pseudo-first-order kinetics, respectively. During the amendment process, the rare microorganisms *Sericytochromatia*, *Parasegetibacter*, *Fusarium* and *Cystobasidium* effectively mediated tailings multifunctionality. The properties of external carbon sources were the primary factors regulating the carbon cycle in tailings via *pgk*, *cellulase*, and *pdh*, while the tailings microenvironment was the key variable directly driving this cycle through *cs*, *g6pd*, *ms*, and *cat*.

## Environmental implications

Iron tailings pose widespread concerns because of their serious threat to ecological security. The present study compared the effects of different types of organic matter on the microenvironment of iron tailings. The experimental results confirmed that easily decomposable amendments markedly improved the organic matter decomposition potential of tailings and promoted an exponential increase in the multifunctional index of tailings. Gene analysis revealed that rare microorganisms, rather than dominant taxa, drove tailings multifunctionality and regulated carbon cycling processes. Organic matter application significantly enhanced the abundances of *pgk*, *cellulase*, *pdh*, *cs*, *g6pd*, *ms*, and *cat* genes, which contributed to the regulation and maintenance of carbon cycling functions in tailings. These findings demonstrated that the application of humic substances or agricultural waste amendments can improve tailings multifunctionality and effectively drive carbon metabolism in iron tailings. These findings suggest that it would be advantageous to harness exogenous organic carbon amendment in promoting ecological restoration and functional improvement of iron tailings. From these, large-scale field trials will be necessary in the near future to further optimize the practical application of exogenous carbon sources for tailings remediation, providing a theoretical basis for developing field feasible and popularized tailings improvement technologies.

## Data Availability

The datasets generated and/or analysed during the current study are available in the NCBI BioProject repository, PRJNA1428724.
